# Study on the Antifibrotic Effects of Recombinant Shark Hepatical Stimulator Analogue (r-sHSA) *in Vitro* and *in Vivo*

**DOI:** 10.3390/md13085201

**Published:** 2015-08-18

**Authors:** Ying Wang, Xiaoyuan Zhang, Yang Yang, Xiaohong Yang, Boping Ye

**Affiliations:** School of Life Science and Technology, China Pharmaceutical University, Nanjing 210009, China; E-Mails: wstarty@gmail.com (Y.W.); oluckyo@126.com (X.Z.); yy05429@126.com (Y.Y.); rainbowyank@163.com (X.Y.)

**Keywords:** r-sHSA, hepatic fibrosis, antifibrosis, carbon tetrachloride, hepatic stellate cells

## Abstract

Hepatic fibrosis is an effusive wound healing process, characterized by an excessive deposition of extracellular matrix (ECM), as the consequence of chronic liver injury of any etiology. Current therapeutic repertoire for hepatic fibrosis is limited to withdrawal of the noxious agent, which is not always feasible. Hence, in this article, the antifibrotic effects and possible mechanisms of r-sHSA, a recombinant protein with hepatoprotection potential, were investigated. Using NIH/3T3 (mouse embro-fibroblast cell line), skin fibroblasts (human skin fibroblasts, SFBs) and HSC-T6 (rat hepatic stellate cell line), the *in vitro* effect of r-sHSA was evaluated by measuring the expression levels of alpha-1 Type I collagen (Col1A1) and α-smooth muscle actin (α-SMA). It turned out those fibrosis indicators were typically inhibited by r-sHSA, suggesting its capacity in HSCs inactivation. The antifibrotic activity of r-sHSA was further investigated *in vivo* on CCl_4_-induced hepatic fibrosis, in view of significant improvement of the biochemical and histological indicators. More specifically, CCl_4_-intoxication induced a significant increase in serological biomarkers, e.g., transaminase (AST, ALT), and alkaline phosphatase (ALP), as well as disturbed hepatic antioxidative status; most of the parameters were spontaneously ameliorated to a large extent by withdrawal of CCl_4_, although the fibrotic lesion was observed histologically. In contrast, r-sHSA treatment markedly eliminated fibrous deposits and restored architecture of the liver in a dose dependent manner, concomitantly with the phenomena of inflammation relief and HSCs deactivation. To sum up, these findings suggest a therapeutic potential for r-sHSA in hepatic fibrosis, though further studies are required.

## 1. Introduction

In animals, the liver is a vital organ characterized by its crucial roles in maintaining metabolic homeostasis. However, the unique position of the liver also confers vulnerability to a wide variety of insults and injury [1], which induces a high prevalence of hepatic diseases. In many cases, following chronic or repeated liver insults, there is progressive fibrosis, irrespective of cause [2]. As a feature of most types of chronic liver diseases, hepatic fibrosis is a reversible wound-healing response, characterized by excessive deposition of a fibrotic matrix rich in in type I collagen, named extracellular matrix (ECM) [3,4]. The state tends to progress, leading to the destruction of the liver architecture by the fibrous tissue septa and regenerative nodules, ultimately terminating in cirrhosis, with increased risk of chronic liver failure or carcinoma [5]. Liver fibrosis, and especially cirrhosis, has recently been reported to be associated with significant morbidity and mortality [6]. Thus, there is a considerable imperative to develop antifibrotic strategies that are applicable to liver fibrosis [7].

The etiologies of chronic liver injury and fibrogenesis have wide geographic distribution and high diversity, including virus, toxins (alcohol, chemicals, iron overload), parasitemia, and metabolic diseases, *etc.* [8]. It is widely accepted that the activation and amplification of hepatic stellate cells (HSCs) play a crucial role in hepatic fibrosis, in terms of the pathogenesis [4]. In consequence of the insult on hepatocytes, increased steady-state levels of reactive oxygen species (ROS) and lipid peroxidation products or inflammatory mediators (e.g., tumor necrosis factor alpha (TNF-α), transforming growth factor beta 1 (TGF-β1)) trigger the activation of HSCs and the loss of retinoids storage [9,10]. During the activation process, HSCs undergo significant morphological and functional changes, which is very reliably indicated by the expression of α-smooth muscle actin (α-SMA), leading to the acquisition of a myofibroblast-like cell phenotype and ultimately to excessive production of collagen [11,12].

Recombinant shark hepatical stimulator analogue (r-sHSA), is a recombinant protein encoded by a novel cDNA sequence, which was cloned from the regenerative hepatic tissues of shark, *Chiloscyllium plagiosum* (GeneBank Access number: AY099492) [13,14]. In previous studies, the significant hepatoprotective effects of r-sHSA have been reported on acute liver injury induced by carbon tetrachloride (CCl_4_) or concanavalin A (Con-A) in mice. In an approximately dose-dependent manner, r-sHSA alleviates acute liver injury by decreasing serum aminotransferase activities, ameliorating hepatocyte necrosis and reducing inflammatory cell infiltration, which might be attributed to the effects of antioxidant and anti-inflammatory [15,16]. Those results implies an inhibitory potential of r-sHSA on hepatic fibrosis, in view of its effects on hepatoprotection, immunoregulation, and fibroblast suppression. In this study, the *in vitro* and *in vivo* effects of r-sHSA on development of hepatic fibrosis are evaluated.

## 2. Results

### 2.1. r-sHSA Inhibits Fibroblasts in Vitro

**Figure 1:**
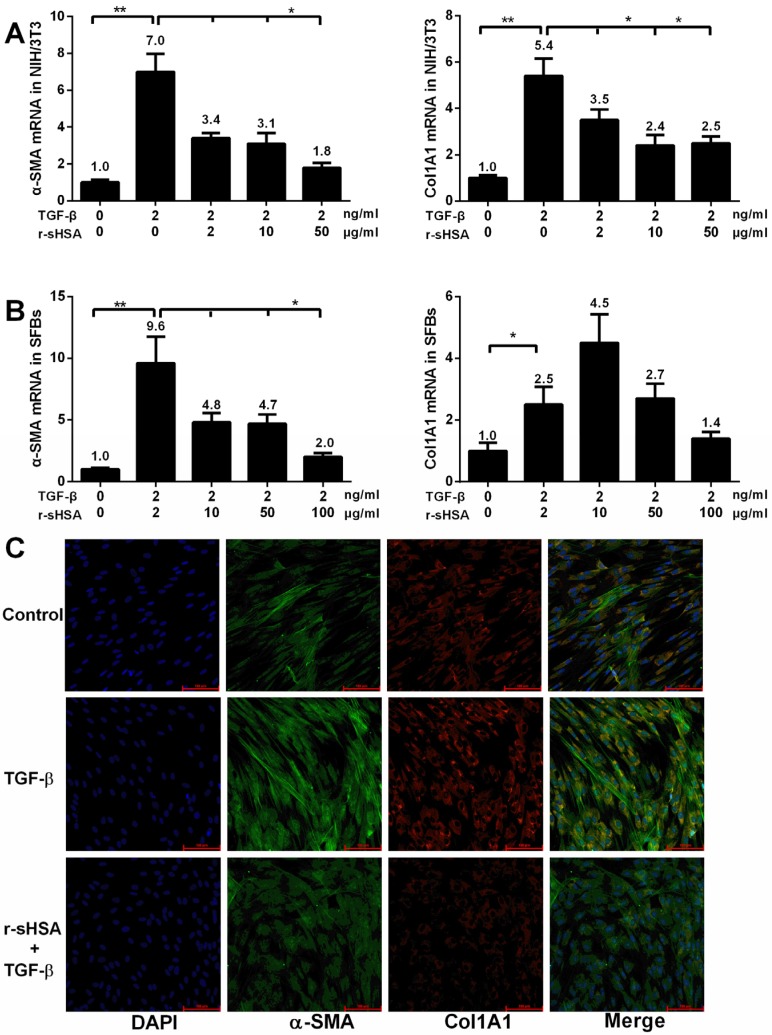
r-sHSA inhibits fibroblasts *in vitro* by measuring Col1A1 and α-SMA expression. Confluent NIH/3T3 and human skin fibroblasts (SFBs) were exposed to TGF-β1 (2 ng/mL) and/or r-sHSA at indicated concentration. Total RNA was subjected to qRT-PCR. The results represent the means ± SD of triplicate determinations. The inhibitory effects of r-sHSA on Col1A1 and α-SMA mRNA expression were demonstrated in NIH/3T3 (**A**) and SFBs (**B**). SFBs were pre-treated with r-sHSA (20 μg/mL) for 24 h, followed by TGF-β1 (4 ng/mL) for 6 h. Then the cells were immunostained with antibodies to α-SMA (green), collagen I (red) or DAPI (blue), and examined by confocal microscopy. Representative immunofluorescence photomicrographs. Original magnification, ×20 (**C**). α-SMA, α-smooth muscle actin; DAPI, 4′,6-diamidino-2-phenylindole. * *P* < 0.05, ** *P* < 0.01 denotes significant difference.

To investigate the effects of r-sHSA on fibrogenesis *in vitro*, NIH/3T3 (mouse embro-fibroblast cell line) and primary cultures of human skin fibroblasts (SFBs), established from neonatal foreskin biopsies, were employed to examine on the changes of gene expression by quantitative reverse transcript-PCR (qRT-PCR) and immunocytochemistry. Amplification was performed using specific primers (Table 1) and fluorescent dye method. It turned out that the expression levels of typical indicators for fibrosis, alpha-1 Type I collagen (Col1A1) and α-smooth muscle actin (α-SMA), were significantly elevated, followed by the transforming growth factor beta (TGF-β, 2 ng/mL) treatment. However, after r-sHSA intervention, a dose-dependent inhibition of Col1A1 and α-SMA were demonstrated, with an apparent reduction (Figure 1A,B). Moreover, on a protein level, potent inhibition of Type I collagen and α-SMA by r-sHSA was confirmed by immunostaining in SFBs (Figure 1C).

**Table 1:** Primers used for qRT-PCR analysis of gene expression.

Gene Name	Forward (5′-3′)	Reverse (5′-3′)	Accession Number
Mouse 18S rRNA	CCCCATGAACGAGGGAATT	GGGACTTAATCAACGCAAGCTT	NM_08324.3
Mouse COL1A1	TGGTCCCAAAGGTTCTCCTGGT	TTAGGTCCAGGGAATCCCATCACA	NM_053304.1
Mouse α-SMA	CTATAACCGGAACTTCTGCCAG	CTGCTCTGTGTCAGGTGTG	NM_031004.2
Human COL1A1	GCTGGTGTGATGGGATTC	GGGAACACCTCGCTCT	NM_000088
Human α-SMA	CAGGGCTGTTTTCCCATCCAT	GCCATGTTCTATCGGGTACTTC	NM_001613
Human 18S rRNA	CCCCATGAACGAGGGAATT	GGGACTTAATCAACGCAAGCTT	NR_003286

### 2.2. r-sHSA Attenuates TGF-β-Induced HSCs Activation

**Figure 2:**
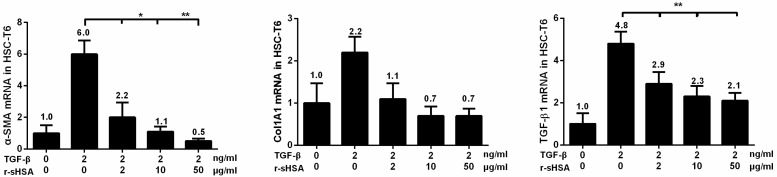
r-sHSA mediates suppression of HSCs activation induced by TGF-β1. Confluent HSC-T6 cells were incubated with the indicated concentrations of r-sHSA and TGF-β1 (2 ng/mL). Total RNA was harvested and subjected to qRT-PCR, to analyze mRNA levels of α-SMA, Col1A, and TGF-β1. The results represent the means ± SD of triplicate determinations. α-SMA, α-smooth muscle actin; Col1A, alpha-1 type I collagen; TGF-β1, transforming growth factor beta 1. * *P* < 0.05, ** *P* < 0.01 denotes significant difference.

In view of the pivotal role of HSCs in orchestrating fibrogenesis, it is of interest to evaluate whether the antifibrotic effects of r-sHSA could be due to HSCs inactivation. For this purpose, HSC-T6, an immortalized rat hepatic stellate cell line, was pretreated with r-sHSA followed by incubation with TGF-β. The results of qRT-PCR showed that in the activated cells elicited by TGF-β, r-sHSA caused a dose-dependent attenuation of α-SMA, collagen and endogenous TGF-β1 gene expression, with an almost 50% reduction at low dosage (Figure 2). Perceivably, in view of the symbolic role of α-SMA in HSCs activation, the antifibrogenesis effects of r-sHSA might be ascribed, at least partially to HSCs deactivation.

### 2.3. r-sHSA Ameliorates Liver Injury Induced by CCl_4_

Chemically induced liver injury was indicated by elevated levels of serum enzymes [aspartate aminotransferase (AST), alanine aminotransferase (ALT), alkaline phosphatase (ALP)] and pathological changes, characterized by hepatocellular necrosis, inflammation, and steatosis [17,18]. A series of serum indexes, including AST, ALT, and ALP, were measured to evaluate the quality of liver function in model animals. As shown in Table 2, CCl_4_ treatment significantly increased AST, ALT, and ALP levels compared to the normal group (*P* < 0.01). In the CCl_4_ control group, observed for the spontaneous regression of fibrosis for two weeks, the pathological damnification was noticeably alleviated. The trend was further highlighted by r-sHSA administration. Of note, higher doses of r-sHSA (60 and 120 μg/kg), decreased ALP activity approximately to normal values.

Moreover, the oxidation resistance of r-sHSA was reflected from the level of malonaldehyde (MDA), glutathione (GSH), and total antioxidant capacity (TAOC). The data indicated, as compared to normal mice, CCl_4_ treatment led to a noticeable rise of hepatic MDA (a marker of lipid peroxidation levels) and depletion of GSH and TAOC, suggesting the impairment of hepatic antioxidant capabilities [19]. After two-week recovery, those indices improved markedly in CCl_4_ control group. Even more apparently, r-sHSA treatment restored hepatic GSH and TAOC levels, as well as ameliorated hepatic MDA contents. (Table 3)

### 2.4. r-sHSA Alleviates CCl_4_-Induced Hepatic Fibrosis

**Figure 3:**
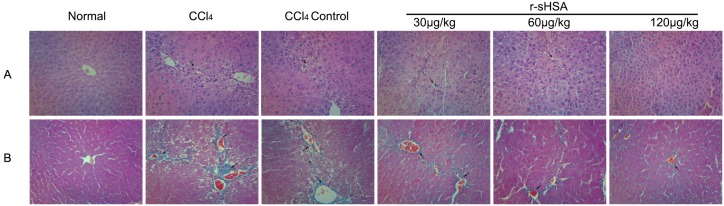
r-sHSA protects liver from fibrotic lesion induced by CCl4. (**A**) Histological images of mice livers stained with H & E (Original magnification ×200). Arrows show injured hepatocytes with steatosis; (**B**) The histopathologic detection of collagen in the livers by Mallory trichrome stain (Original magnification ×200). Collagen fibers of the connective tissues are identified by their blue color (arrow).

The CCl_4_-induced fibrogenesis and r-sHSA effects were evaluated through histopathology (Figure 3). In the liver sections of CCl_4_-treated mice, adipose degeneration of hepatocytes, broad wall thickening of central venous, severe fibrous hyperplasia, and inflammatory cell infiltration were observed. The morbid events were retarded via spontaneous recovery to some extent, however, the fibrotic characteristics were apparent in CCl_4_ control group, with visible steatosis, inflammation, collagen deposition, and fibrous septa. Against the hepatocyte damages, the treatment with r-sHSA showed a dose-dependent effect, with significant improvement in higher dosages. Consistently, as shown in Table 4, after CCl_4_ treatment, fibrosis scores of livers were significantly higher than normal animals. The fibrosis score partly fell in CCl_4_ control group; however, compared with the r-sHSA treat groups, it was still at an elevated level.

The events were also confirmed via biochemical analyses. Serological or histological biomarkers, such as hyaluronic acid (HA), tissue inhibitor of metalloproteinases-1 (TIMP-1), hydroxyproline (Hyp) and collagen III, were engaged to the estimation of fibrogenesis and reversibility (Table 2). Those fibrosis indicators substantially ascended in the CCl_4_ group, to 2–3 times those in the normal mice (*P* < 0.01). The next two-week spontaneous resolution significantly decreased the levels of these markers (*P* < 0.05). However, r-sHSA treatment further accelerated the recovery, evidently performing the antifibrotic effects at higher dosage (60–120 μg/kg), as compared with the CCl_4_ control group (*P* < 0.05).

**Table 2:** Biochemical parameters of liver injury and fibrosis.

	Normal	CCl_4_	CCl_4_ control	r-sHSA		
				30 μg/kg	60 μg/kg	120 μg/kg
**ALT (IU/L)**	40.19 ± 14.47 **^++^	166.80 ± 37.12 ^++^	97.47 ± 32.61 **	89.57 ± 16.72 **	74.37 ± 32.39 **	72.53 ± 27.77 **
**AST (IU/L)**	65.75 ± 9.86 **^++^	244.80 ± 61.55 ^++^	107.46 ± 32.37 **	89.24 ± 11.64 **	78.85 ± 29.03 **^+^	75.35 ± 23.07 **^+^
**ALP(IU/L)**	38.33 ± 3.20 **	57.71 ± 6.97 ^+^	41.06 ± 10.32 *	40.81 ± 6.83 **	43.54 ± 4.85 **	40.27 ± 2.41 **
**HA(ng/mL)**	74.17 ± 10.70 **^++^	133.10 ± 15.31 ^++^	101.10 ± 9.61 **	95.30 ± 9.73 **	83.60 ± 4.57 **^++^	85.66 ± 7.51 **^+^
**TIMP-1 (ng/mL)**	4.68 ± 0.44 **^+^	8.53 ± 0.71 ^++^	5.63 ± 0.67 **	6.01 ± 0.84 **	5.49 ± 0.52 **	4.59 ± 0.41 **^+^
**Hyp (μg/g liver)**	136.92 ± 14.43 **^++^	487.39 ± 31.83 ^+^	402.99 ± 70.56 *	387.07 ± 37.46 **	312.98 ± 47.06 **^+^	199.02 ± 18.41 **^++^
**Collagen III (ug/g protein)**	0.35 ± 0.06 **^++^	0.94 ± 0.18 ^+^	0.71 ± 0.11 *	0.55 ± 0.14 **	0.49 ± 0.05 **^+^	0.47 ± 0.11 **^+^

Comparison of serological markers for liver function and fibrosis, including aspartate aminotransferase (AST), alanine aminotransferase (ALT), alkaline phosphatase (ALP), hyaluronic acid (HA) and tissue inhibitor of metalloproteinases-1 (TIMP-1). All results represent means ± SD for 12 mice. * *P* < 0.05, ** *P* < 0.01 *vs.* CCl_4_ group. ^+^ *P* < 0.05, ^++^ *P* < 0.01 *vs.* CCl_4_ control group.

**Table 3:** Hepatic indicators of oxidative stress.

	Normal	CCl_4_	CCl_4_ control	r-sHSA		
				30 μg/kg	60 μg/kg	120 μg/kg
**MDA (nM/mg protein)**	5.03 ± 1.59 *	7.88 ± 1.34 ^+^	5.94 ± 1.20 *	5.73 ± 0.82 *	4.74 ± 0.86 **	5.13 ± 0.83 **
**GSH (mg/g protein)**	3.80 ± 0.56 **	2.66 ± 0.30 ^+^	3.30 ± 0.53 *	4.49 ± 1.20 **	6.11 ± 1.21 **^++^	6.89 ± 0.87 **^++^
**TAOC (U/mg protein)**	1.57 ± 0.26 **	1.02 ± 0.22 ^+^	1.37 ± 0.13 *	1.49 ± 0.25 **	1.67 ± 0.41 *	1.67 ± 0.22 *^+^

Each value represents means ± SD for 12 mice. * *P* < 0.05, ** *P* < 0.01 *vs.* the CCl_4_ group. ^+^ *P* < 0.05, ^++^ *P* < 0.01 *vs.* the CCl_4_ control group.

**Table 4:** Pathological evaluation in views of fibrosis scores.

	Normal	CCl_4_	CCl_4_ control	r-sHSA		
				30 μg/kg	60 μg/kg	120 μg/kg
**Fibrosis score**	0 ± 0	3.42 ± 0.76 *^++^	2.33 ± 0.94 **	2.00 ± 0.82 **	1.83 ± 0.80 **	0.83 ± 0.69 **^++^
**Hepatocyte necrosis**	0 ± 0	1.67 ± 0.62 *^+^	1.08 ± 0.64 *	1.00 ± 0.71 *	0.58 ± 0.49 **	0.17 ± 0.37 **^++^

Each value represents means ±SD for 12 mice. * *P* < 0.05, ** *P* < 0.01 *vs.* the CCl_4_ group. ^+^ *P* < 0.05, ^++^ *P* < 0.05 *vs.* the CCl_4_ control group.

### 2.5. r-sHSA Inhibits HSCs Activation and Inflammation

**Figure 4:**
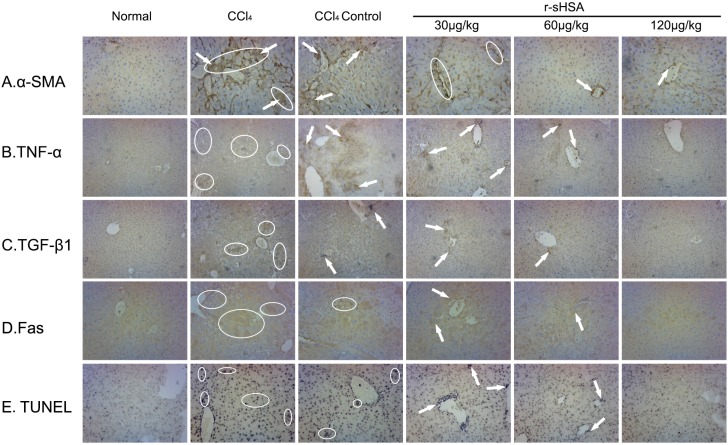
r-sHSA inhibits fibrotic factors and hepatocytes apoptosis. The expression and specific tissue distribution of α-SMA (**A**), TNF-α (**B**), TGF-β1 (**C**) and apoptotic cells by TUNEL (**D**) and Fas (**E**) with immunohistochemical stain. Original magnification ×200 for **A**, **B**, **D**, and ×100 for **C**, **E**. Representative immunopositive area are circled and indicated by arrows, which are identified by brown/violet color.

With regard to the distribution of α-SMA-positive fibrogenic cells, *in vivo* influence on HSCs activation of r-sHSA was assessed (Figure 4A). In normal livers, α-SMA immunopositivity was restricted to arterial tunica media, central venous and arterial wall, but remained negative in other regions. CCl_4_ strongly induced perisinusoidal α-SMA expression, corresponding to activated HSCs, which connected between themselves via thin, “bridging” immunopositivity, with distorted lobular architecture. Then, due to spontaneous reversal, perisinusoidal immunopositivity was withdrawn, yet clearly presented in hepatocytes located at the scar-parenchyma interface. By contrast, r-sHSA efficiently inhibited HSCs activation, with sporadic α-SMA immunopositive cells at the dose of 60 μg/kg. Following the increase dosage increase (120 μg/kg), the staining pattern shrank, even close to normal.

In addition, the inflammatory response during hepatic fibrogenesis was estimated by changes of proinflammatory factors, TNF-α (Figure 4B) and TGF-β1 (Figure 4C), which provided additional evidence for therapeutic effects of r-sHSA. Briefly, normal livers had moderate TNF-α/TGF-β1 expression in the perilobular areas, whereas CCl_4_ substantially initiated tissue inflammation, characterized by wide immunopositivity in hepatic sinusoid, portal tracts, fibrous septa, and necrotic hepatocytes. In the CCl_4_ control group, immunopositivity persisted with some degree of improvement, followed by the partial resolution of fibrous structure. However, treated by r-sHSA, immunopositive deposits were sporadically distributed in inflammatory cells infiltration in the portal area and hepatic sinusoidal cytoplasm, with a significant decrease at higher dose.

### 2.6. r-sHSA Moderates Hepatocyte Apoptosis

The apoptosis of parenchymal and nonparenchymal cells were represented by positive staining of TUNEL (Figure 4D) and Fas immunopositivity (Figure 4E). Hepatotoxicity of CCl_4_ markedly caused hepatocellular apoptosis, featured by wide distribution of positive staining for TUNEL and Fas protein, round fiber hyperplasia, pseudolobuli and portal inflammation. The case was partially improved but continued, after spontaneous recovery. However, apoptotic cells decreased significantly after r-sHSA treatment, which showed a dose-effect relationship.

## 3. Discussion

In this work, the antifibrotic effects of r-sHSA, a recombinant protein with hepatoprotective potential, were investigated *in vitro* and *in vivo* respectively. Consistent to previous hypothesis, the results showed that r-sHSA could inhibit different fibroblasts, demonstrated by the descending collagen and α-SMA. Furthermore, *in vivo* study also suggested that r-sHSA could significantly protect liver against fibrosis induced by CCl_4_, on account of the histopathologic examination and improvement on fibrotic indexes.

As an important experimental hepatotoxicant, CCl_4_ is proposed to cause fatty infiltration and centrilobular necrosis in liver, as well as destruction of microsomal cytochrome P-450 [20]. The major mechanism of CCl_4_ hepatoxicity, derives from its active metabolites generated by a cytochrome P-450 dependent step, which can bind covalently with membrane structures, thereby inducing lipid peroxides [21,22]. Increased lipid peroxidation subsequently causes the initiation of oxidative stress, which plays an eminent role in the pathogenesis of liver diseases, by mediating inflammatory process, cell apoptosis and tissue injury [23,24]. In our study, following continuous exposure to CCl_4_, a dramatic ascent was observed in a series of biomarkers (ALT, AST, ALP, HA and TIMP-1) in model mice; oxidative stress also was demonstrated by alteration of relevant parameters, such as MDA, GSH, and TAOC, which indicated the status of oxidative attack on hepatocytes and tissue [25]. However, the pathological condition could be modified by administration of r-sHSA, implying its beneficial effects on hepatocellular integrity, in which the natural antioxidant mechanisms might be involved [26].

Being a highly dynamic process, liver fibrosis is now known as reversible in human and animal models, following control of the noxious stimulus [27,28,29], implying recovery with tissue remodeling is possible [30]. Spontaneous regression actually was observed in our research: after discontinuation of CCl_4_ for two weeks, the above-mentioned biochemical parameters in CCl_4_ control mice indicated essential melioration of oxidative stress and hepatocellular damage, albeit slightly less effective than the groups treated with r-sHSA. Additionally, previous studies stated that the classical biomarkers, such as serum aminotransferase levels, do not necessarily reflect the degree and progression of chronic liver damage [31,32], since a complex ROS-adaptive response has developed in mammals, to prevent most oxidative damage from ever occurring [33,34]. Hence, histopathological assessments were further analyzed to monitor the stage of fibrotic process, as well as the ECM deposition or removal. Consistently, morphological studies showed that fibrotic lesion of the liver typically involved in CCl_4_-intoxicated group, besides an accumulation of ECM, which characterized the presence of fibrosis [35]. In contrast, the pathologic status ameliorated modestly in CCl_4_ control group, with a shrinkage in α-SMA immunopositive area and collagen deposition, after spontaneous recovery for two weeks, yet inferior to the therapeutic effects of r-sHSA, which also manifested the potency of r-sHSA on fibrosis reversal.

Liver fibrosis represents a common response to chronic hepatic damage in various diseases, which results in ECM remodeling and scar tissue formation, characterized by the excessive deposition of collagen [36]. For decades, the evidence for the pivotal role of HSCs in liver fibrogenesis is compelling, which orchestrates the pathogenesis, with cooperation of diverse cellular and molecular basis [37]. Generally, HSCs undergo transdifferentation into a myofibroblastic phenotype, a process termed activation, associated with loss of the retinoid droplets and expression of α-SMA. Activated HSCs show de novo fibrogenic properties, including proliferation and accumulation in areas of hepatocytes necrosis, secretion of proinflammatory cytokines and chemokines, and synthesis of a large panel of matrix proteins and of inhibitors of matrix degradation, leading to progressive scar formation [38,39]. Therefore, from the practical perspective, those key elements might lay the basis for rational design of antifibrogenic strategies.

In fact, the antifibrotic activity of r-sHSA likely involves such crucial mechanisms. As the principal collagen-producing cells [40], HSCs activation remains the major paradigm pathway in hepatic fibrogensis [41]. Using fibrotic mice model induced by CCl_4_, increased α-SMA perisinusoidal immunopositivity, as well as the deposition of collagen in damaged hepatic areas, indicated activated HSCs are responsible for the fibrosis seen in the CCl_4_-intoxicated mice. Nevertheless, α-SMA positive expression, was observed to decrease after r-sHSA treatment, accompanied by the descending collagen content. Similarly, a marked fall was observed in the mRNA expressions of α-SMA and collagen I, in two kinds of fibroblasts and HSC-T6 cells, as witness to that case *in vitro*. Those results demonstrated that r-sHSA could inhibit HSCs activation, which paves the way for fibrosis recovery.

From another aspect, the crucial role of HSCs activation in fibrotic process leads to extensive concerns on numerous signals, of which HSCs serve as both the principal source and the target [42]. Hitherto, the dominant cytokines and chemokines have been enumerated to constitute a major class of mediators responsible for hepatic fibrogenesis modulation [43,44,45]. Among them, inflammatory cytokines like TNF-α, or fibrogenic growth factors, e.g., TGF-β1, generally recognized as being of great importance, are both are profoundly implicated in tissue repair and matrix accumulation [46]. Essentially, the etiological role of TGF-β1 in fibrogenesis has been clarified in numerous experimental and human fibrotic disorders [47]: as a dominant stimulus to ECM production by HSCs, the detrimental effects of TGF-β1 involve a range of cellular and molecular responses, leading to perpetuation of HSC activation, parenchymal cell apoptosis, as well as matrix synthesis [48,49,50]. Moreover, induced by TGF-β1, the collagenolytic activity was dramatically decreased, accompanied by elevated specific tissue inhibitors of the metalloproteinase family (TIMPs), resulting in an imbalance between matrix synthesis and breakdown, which undoubtedly worsens the ongoing fibrotic development [39,51]. In this study, after chronic exposure to CCl_4_, apparent deposits of TGF-β1 present in necrotic areas, suggests an active producing of this profibrotic cytokine, which predictably lead to consequent HSCs activation and ECM accumulation. Concomitantly with the resolution of fibrosis, r-sHSA treatment diminished TGF-β1 expression by comparison.

As a pleiotropic cytokine, TNF-α participates in a variety of physiological and pathophysiological processes [52]. It has been reported TNF-α could regulate gene transcription, consequently inducing a variety of molecular signaling pathways, which involve the pathologic inflammatory and fibrotic milieu in liver [53]. Additionally, the detrimentally profibrogenic effect of TNF-α could be associated with the induction of collagen deposition and hepatocyte apoptosis [54]. Comparably, in our research, omnipresent immunopositivity in the CCl_4_ group suggested a high levels of TNF-α, accompanied by obvious steatosis and oxidative lesion, which was sustained in CCl_4_ control group with moderate improvement. The therapeutic activity of r-sHSA was contrastively associated with substantial decrease in TNF-α production, concomitantly with the histological improvement in the livers.

Increasingly as a nexus, apoptosis is associated with liver disease through which many key pathways converge. As to hepatic fibrogenesis, a pervasive but complex interplay takes place among those cellular and molecular bases, such as HSCs activation, peroxidation, inflammatory mediators, as well as apoptosis [55]. In order, following liver insults, damaged parenchymal cells release inflammatory and fibrogenic mediators, which promote hepatocyte apoptosis and the recruitment of inflammatory cells. Engulfment of the apoptotic bodies enhances profibrogenic and proapoptotic effects via gene expression alteration, which further sustain inflammatory response and HSC activation in a feed-forward-loop process [3,55,56,57]. Identical to the pathogenic mechanism, CCl_4_ intoxication was associated with a high proportion of hepatocyte apoptosis, characterized by ubiquitous immunopositivity of TUNEL and Fas protein, which coincidently matched the enhancement of TNF-α. By contrast, r-sHSA decreased hepatocyte apoptosis, along with an amelioration of tissue injury, which might suggest its involvement in protection of hepatic parenchyma from inflammatory and fibrotic disorders.

## 4. Experimental Section

### 4.1. Preparation of r-sHSA

r-sHSA was expressed in *Escherichia coli* BL21(DE3) with the recombinant vector pET-28a-sHSA and purified as previously described [14]. Briefly, the engineered bacteria of r-sHSA were cultured overnight in the optimized liquid medium [58]. Induced by 0.5 mM IPTG for 6 h, cells from high-density fermentation were harvested by centrifugation, resuspended in 50 mM Tris-HCl (pH 9.0) and ultrasonicated. The inclusion body was prepared by continuous wash, denaturation, and renaturation. Chromatographically purified by Q Sepharose Fast Flow (GE Healthcare, Uppsala, Sweden), proteins then were cleaved by thrombin (Novagen, San Diego, CA, USA) and separated by Heparin Sepharose media (GE Healthcare, Uppsala, Sweden) from the redundant His-tag. The final products were concentrated by ultrafiltration with a purity up to 97.8%, determined by HPLC.

### 4.2. Cells and Cell Culture

Primary cultures of human skin fibroblasts (SFBs) were kindly provided by John Varga (Northwestern University, Chicago, IL, USA),which were established from neonatal foreskin biopsies by previously described explant techniques [59]. SFBs were maintained in a 5% CO_2_ atmosphere at 37 °C in DMEM medium, 10% fetal bovine serum (FBS), 1% vitamins, 2mM l-glutamine and 1% penicillin/streptomycin (BioWhittaker, Walkersville, MD, USA). For all experiments, SFBs were studied between passages 4 and 8 [60].

Another two kinds of cell line, mouse embryo fibroblast NIH/3T3 and rat hepatic stellate cell HSC-T6 were obtained from Type Culture Collection of the Chinese Academy of Sciences (Shanghai, China). Cells were cultured at 37 °C in 5% CO_2_ in DMEM growth medium, supplemented with 10% FBS, 2mM l-glutamine and standard antibiotics (Hyclone, Logan, UT, USA) [61].

### 4.3. Antifibrotic Assay in Vitro

The *in vitro* effects of r-sHSA on the fibrosis development were investigated on multiple cell types. For each type, a total of 6 × 10^5^ cells were seeded in a 12-well plate and allowed to 90% confluency, and then replaced with fresh medium containing 0.1% BSA. After serum-starvation for 24 h, cells were treated with TGF-β1 (2 ng/mL, R&D system, Minneapolis, MN, USA) in the absence or presence of r-sHSA at indicated various concentration for 24 h. Following exposure to TGF-β1 for 6 h, cells were harvested for measuring the expressions of endogenous TGF-β1, α-SMA and alpha-1 type I collagen (Col1A1) by quantitative RT-PCR analysis (qRT-PCR) [62].

### 4.4. Quantitative Reverse Transcriptase Polymerase Chain Reaction (qRT-PCR)

At the end of treatment, cell lysates were subjected to RNA isolation using Qiagen RNeasy^®^ plus kit (Qiagen, La Jolla, CA, USA). First strand cDNA was synthesized from 2 μg of total RNA using the M-MLV Reverse Transcriptase (Promega, Madison, WI, USA) and used as a template for qRT-PCR. PCR amplifications were performed using the primer pairs for qRT-PCR (Table 1) in 1× Power SYBR mix (Applied Biosystems, Foster City, CA, USA) on a ABI 7300 Thermocycler (Applied Biosystems, Foster City, CA, USA). Relative quantification (RQ) of each gene expression was calculated as previously described [63], according to comparative Ct method using the formula: RQ = 2^−ΔΔCt^, with E = 100% and the expression of 18S rRNA as the endogenous calibration.

### 4.5. Confocal Immunofluorescence Microscopy

SFBs (1 × 10^4^ cells/well) were seeded onto eight-well Lab-Tek II chamber glass slides (Thermo, Waltham, MA, USA) to confluence, and then subjected to serum-starvation for 24 h. Fresh media with r-sHSA (20 ug/mL) were added and the incubations continued for 24 h, then treated with TGF-β1 (4 ng/mL) for another 24 h. At the end of the experiments, cells were fixed, permeabilized, and incubated with primary antibodies to Type I collagen (Southern Biotech, Birmingham, AL, USA) or to α-SMA (Sigma, St. Louis, MO, USA) at 1:500 dilution. Cells were then washed with PBS and incubated with secondary antibodies at 1:500 dilution (Alexa Fluor 488 and 594, Invitrogen, Grand Island, NY, USA) and viewed under a Nikon A1R Cell Imaging Facility (Nikon, Tokyo, Japan) [62].

### 4.6. Mice and Hepatic Fibrosis Model

Male C57BL/6 mice of clean grade (body weight 18–22 g) were purchased from Comparative medicine center of Yangzhou University (Yangzhou, China), with certification number of SCXK (Su) 2012-0004. The mice were maintained at 12 h light/dark cycle, at constant temperature (20 ± 1 °C) and humidity (50% ± 5%). All animal care and research protocols were performed in compliance with the appropriate laws and animal welfare guidelines, and were approved by the Animal Ethical Committee of China Pharmaceutical University (Nanjing, China).

After five days of acclimation, mice were randomly divided into several groups, with 12 mice in each group (*n* = 12): normal, CCl_4_, CCl_4_ control (observed for spontaneous resolution), r-sHSA groups (30, 60, or 120 μg/kg). Liver fibrosis was induced by subcutaneous administration of CCl_4_ (dissolved in peanut oil) at a dose of 1mL/kg body weight twice-weekly for four consecutive weeks, while normal group received an equal volume of peanut oil. The CCl_4_ group was examined with histopathology, by killing the animals 72 h after the last CCl_4_ injection. For the next two weeks, the CCl_4_ control group was observed for spontaneous resolution. As in the protein-treated groups, r-sHSA was dissolved in saline and administered intraperitoneally (i.p.) at a dose of 30, 60, or 120 μg/kg daily, respectively. The dosages were based on preliminary studies [16]. Whereas mice from the normal and CCl_4_ control groups received vehicle instead. At 24 h after the last administration, whole blood was collected from the orbit of ether anesthetized mice. Then all animals were sacrificed by cervical dislocation, and liver tissue was harvested for biochemical analysis or histopathology examination [52].

### 4.7. Biochemical Analysis

A series of biochemical indexes were measured by spectrophotometric method with the commercially available kits, according to the manufacturers’ instructions on a Model 680 microplate reader (Bio-Rad, Hercules, CA, USA). As biomarkers of liver function, serum enzymes, including alanine aminotransferase (ALT), aspartate transaminase (AST) and alkaline phosphatase (ALP), were measured by Liver function tests kits (Nanjing Jiancheng Bioengineering Institute, Nanjing, China). Levels of serum hyaluronic acid (HA) and tissue inhibitor of metalloproteinases-1 (TIMP-1) were assessed by ELISA kits (Genmed, Shanghai, China).

Hepatic tissues were homogenized in saline and centrifuged at 3500 *g* for 10 min, to collect the supernatant for protein quantitation and multiple analysis on fibrosis indexes. A series of biochemical indicators of inflammation and fibrosis, such as malonaldehyde (MDA), total antioxidant capacity (TAOC), glutathione (GSH), inducible nitric oxide synthase (i-NOS), hydroxyproline (Hyp) and collagen III, were measured using commercially available kits, correspondingly calibrated by the protein concentration (BCA kit, Byotime, Nantong, China). All the procedures were performed at 4 °C [64].

### 4.8. Histopathologic Evaluation

Liver specimens were fixed in 10% formalin, embedded in paraffin, and cut into 4 μm thick sections. Sections for histopathological examination were stained with hematoxylin and eosin (H & E) and Mallory trichrome stain, according to standard procedure.

The extent of fibrosis was examined blindly by a pathologist, numerically graded in the light of the formula of Scheuer [65], with slight modification [66]. Briefly, hepatic fibrosis could be staged as: 0, absence of fibrosis; 1, enlarged, fibrous portal tracts; 2, periportal or portal-portal septa, but intact architecture; 3, fibrosis with architectural distortion; 4, probable or definite cirrhosis. Additionally, hepatocyte necrosis or degeneration severity was graded as [67]: 0, no hepatocyte necrosis or degeneration; 1, focal necrosis or degeneration of hepatocytes (mild, lesion ≤3); 2, multifocal necrosis or degeneration of hepatocytes (moderate, lesion >3); 3, locally extensive or diffuse necrosis or degeneration of hepatocytes (severe). Fibrosis and hepatocyte scores were given after the pathologist had examined three different areas in the tissue slide for each mice.

### 4.9. TUNEL and Immunohistochemical Assay

Apoptotic hepatocytes in sections were detected by terminal dexynucleotidyl transferase (TdT)-mediated dUTP nick end labeling (TUNEL) assay, using the *in situ* cell death detection kit (Roche, Indianapolis, IN, USA), according to the manufacturer’s instructions. TUNEL-positive nuclei were identified by the BCIP/NBT (5-bromo-4-chloro-3-indolyl phosphate/nitro blue tetrazolium) solution as blue/violet staining.

For immunohistochemistry, sections were deparaffinized, rehydrated, and stained using the MaxVision™ IHC kit (Maxim, Fuzhou, China) as described [68]. Briefly, followed by the blockage of normal goat serum, slides were incubated with the antibodies of α-SMA, Fas (1:100; Maxim, Fuzhou, China), TGF-β1 (1:100; Santa Cruz, Dallas, TX, USA) and TNF-α (1:50; Bioworld, St. Louis, MN, USA), respectively, for 1 h at 37 °C. After washing, the tissues were incubated with HRP-polymer secondary antibody for 15 min at 37 °C and then were visualized using diaminobenzidine (DAB) substrate. A negative control was performed by omitting the primary antibody. Stained slides were analyzed under a light microscopy (Olympus, Tokyo, Japan).

### 4.10. Statistical Analysis

Data are presented as means ± SD. Statistical analysis was performed using an unpaired Student’s *t*-test for single, or analysis of variance for multiple, group comparison. In all tests, *P* < 0.05 was considered statistically significant.

## 5. Conclusions

In summary, r-sHSA could inhibit HSCs activation and ECM composition by reducing the expression of α-SMA and collagen *in vitro*. *In vivo*, r-sHSA therapy could result in a complete reversion of progressive liver fibrosis by meliorating fibrotic pathology in the light of indicant and histological levels. As far as the current data is concerned, the antifibrogenic potential of r-sHSA, might involve inactivation of HSC and expression down-regulation of profibtic cytokines (e.g., TNF-α and TGF-β1). The results obtained from present study suggest the therapeutic application of r-sHSA in hepatic fibrosis, although further studies are required.

## Abbreviations

r-sHSA: recombinant shark Hepatical Stimulator Analogue
ECM: extracellular matrix
HSCs: hepatic stellate cells
ROS: reactive oxygen species
α-SMA: α-smooth muscle actin
TNF-α: tumor necrosis factor alpha
TGF-β: transforming growth factor beta
CCl_4_: carbon tetrachloride
SFBs: human skin fibroblasts
FBS: fetal bovine serum
ALT: alanine aminotransferase
AST: aspartate aminotransferase
ALP: alkaline phosphatase
HA: hyaluronic acid
TIMP-1: tissue inhibitor of metalloproteinases-1
Hyp: hydroxyproline
MDA: malonaldehyde
TAOC: total antioxidant capacity
GSH: glutathione
i-NOS: inducible nitric oxide synthase
Col1A1: alpha-1 type I collagen
TUNEL: terminal dexynucleotidyl transferase(TdT)-mediated dUTP nick end labeling
